# Epidermolysis bullosa pruriginosa: A retrospective cohort study highlighting secondary cutaneous amyloidosis and response to Janus kinase inhibition

**DOI:** 10.1016/j.jdin.2026.06.022

**Published:** 2026-07-22

**Authors:** Ping-Chen Hou, Hui-Ching Cheng, Cheng-Lin Wu, Chao-Chun Yang, Sheau-Chiou Chao, Julia Yu-Yun Lee, Chao-Kai Hsu

**Affiliations:** a Department of Dermatology, National Cheng Kung University Hospital, College of Medicine, National Cheng Kung University, Tainan, Taiwan; b Department of Pathology, National Cheng Kung University Hospital, College of Medicine, National Cheng Kung University, Tainan, Taiwan; c Institute of Clinical Medicine, College of Medicine, National Cheng Kung University, Tainan, Taiwan; d International Research Center of Wound Repair and Regeneration (iWRR), National Cheng Kung University, Tainan, Taiwan

**Keywords:** abrocitinib, dermatopathology, epidermolysis bullosa pruriginosa, JAK inhibitor, keratinocytic amyloid, localized cutaneous amyloidosis, whole exome sequencing

*To the Editor:* Epidermolysis bullosa pruriginosa (EBP) is a rare hereditary blistering disorder caused by pathogenic *COL7A1* variants.^1^ Clinically, it is characterized by intensely pruritic, linearly distributed erythematous papuloplaques over the shins.^1^ Rare cases of EBP with concomitant localized cutaneous amyloidosis have been reported,^1^^,^^2^ although the underlying cause and prevalence remain unclear.

We retrospectively reviewed 44 EBP patients in National Cheng Kung University Hospital, Taiwan (July 1988-April 2025) (Supplementary Table I, available via Mendeley at https://data.mendeley.com/datasets/td5cgjfznx/1).^3^ Among the 44 patients, the majority were female and predominantly autosomal dominant dystrophic EBP, with onset ranging from birth to adulthood. Whole exome sequencing (WES) was performed in 27 cases, all carrying pathogenic *COL7A1* variants. The remaining 17 cases lacked genetic testing and were diagnosed by a combination of characteristic clinical manifestations, family history, and/or histopathological findings. Among the 44 EBP cases, 7 (15.9%, 4 genetically confirmed, 3 pathologically diagnosed) showed eosinophilic amorphous materials deposited in the upper dermis, with 6 positive on Congo red stain, confirming amyloid deposition. Cytokeratin 5/6 staining was performed in 4 cases and was positive in all, indicating keratinocytic origin of the amyloids (Supplementary Fig 1, available via Mendeley at https://data.mendeley.com/datasets/td5cgjfznx/1). WES was performed in 3 cases with concomitant amyloidosis to assess for inherited predisposition (Supplementary data provide gene panel, available via Mendeley at https://data.mendeley.com/datasets/td5cgjfznx/1). However, no amyloidosis-related pathogenic variants were identified. Of the 7 cases with concurrent EBP and localized cutaneous amyloidosis, 3 were treated with abrocitinib (100-200 mg/day), resulting in resolution of pruritus and skin lesions flattening (Fig 1, Supplementary Table I, available via Mendeley at https://data.mendeley.com/datasets/td5cgjfznx/1).

**Fig 1:**
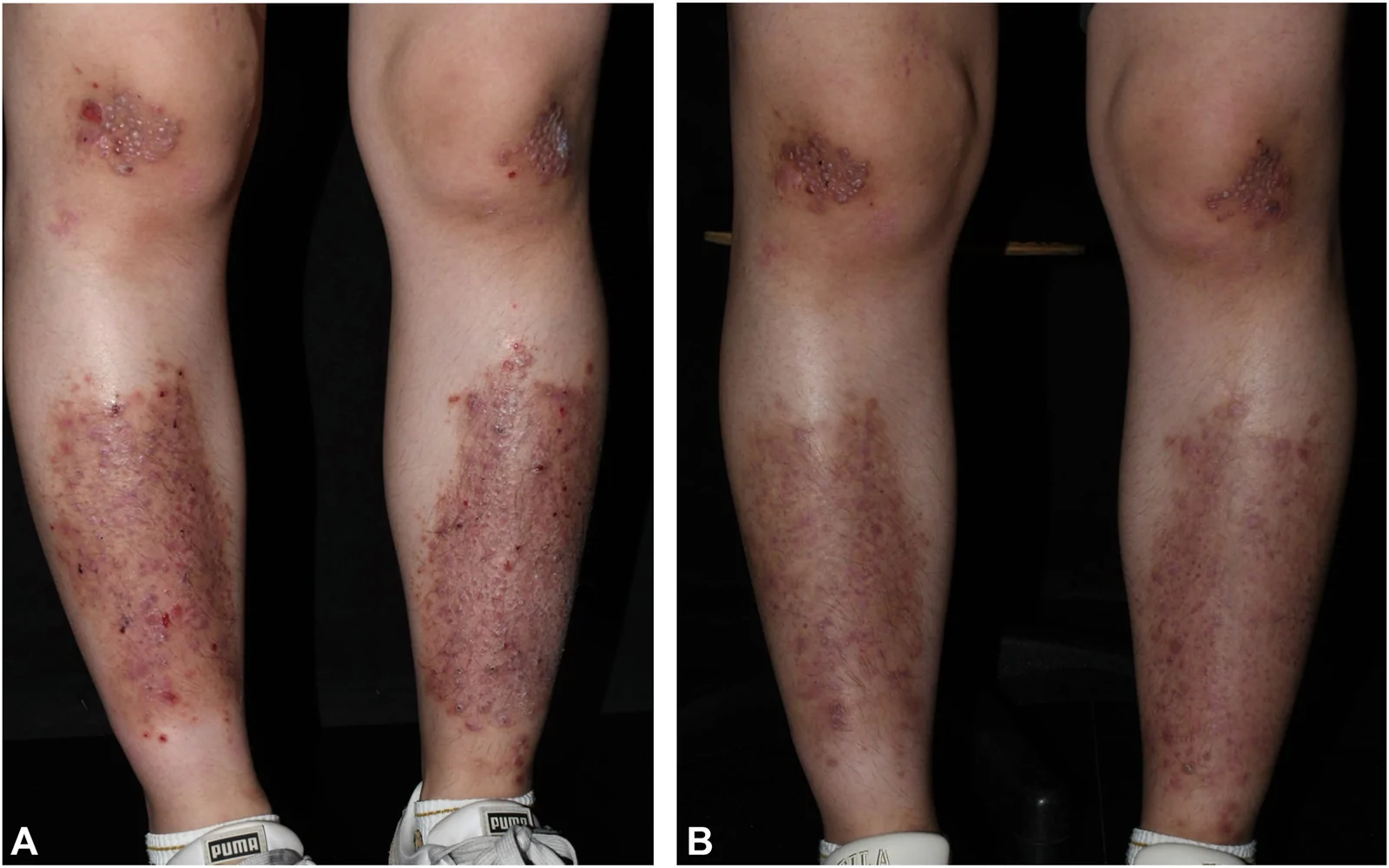
Treatment effect of abrocitinib in an epidermolysis bullosa pruriginosa patient with concomitant localized cutaneous amyloidosis. A 15-year-old female (Patient 1) received abrocitinib (100-200 mg/day) for 4 months and demonstrated less erythema as well as flattening of the skin lesions. Complete resolution of itch was achieved as early as 2 months after the abrocitinib treatment. **A,** Before treatment (itch visual analog score: 4/10). **B,** Four months after treatment (itch visual analog score: 0/10).

Several explanations have been proposed for the coexistence of EBP and localized cutaneous amyloidosis: (1) coincidental occurrence, (2) blister formation causing dermal-epidermal junction (DEJ) damage and keratinocyte degeneration, and (3) chronic scratching causing keratinocyte damage and amyloid formation.^1^^,^^2^ While lichen amyloidosis is relatively common in Asia (7.87/10,000 in Taiwan),^4^ the high rate (15.9%) of concurrent disease in our cohort suggests a noncoincidental association. Recurrent subepidermal blistering in EBP may induce basal keratinocyte degeneration and release of keratin filaments into the papillary dermis. Although all epidermolysis bullosa patients share DEJ fragility, concurrent localized cutaneous amyloidosis has been observed exclusively in EBP—with the exception of 1 case of EB simplex^5^—suggesting that DEJ fragility alone is unlikely to account for this association. WES also excluded an additional inherited predisposition.

EBP is characterized by intractable itch, leading to excessive scratching.^1^ Long-term friction has been implicated in amyloid formation.^2^ Therefore, it is likely that chronic scratching on top of DEJ fragility in EBP may contribute to keratinocyte degeneration and secondary keratinocytic amyloid deposits in the dermis. This is supported by the 3 patients treated with abrocitinib, all of whom reported significant itch reduction accompanied by flattening of the skin lesions. With pruritus adequately controlled, scratching episodes are minimized, which in turn promotes improvement in the lichen amyloidosis–like papules. Interestingly, the amyloid deposit in EBP is situated lower in the dermis as compared to lichen amyloidosis, probably due to dermal fibrosis while etiology remains to be elucidated.

In conclusion, our study underscores the high prevalence of concurrent localized cutaneous amyloidosis in EBP and suggests a potential role for chronic scratching, on a background of DEJ fragility, as a plausible explanation for secondary keratinocytic amyloid deposition. Effective itch control with Janus kinase 1 inhibition not only alleviates pruritus but also improves lichen amyloidosis–like lesions in EBP.

### Declaration of generative AI and AI-assisted technologies in the writing process

None.

## Conflicts of interest

None disclosed.
